# Complete plastome of *Elaeodendron glaucum* (Rottb.) Pers.: genomic resources for Celastraceae systematics

**DOI:** 10.3389/fpls.2025.1649473

**Published:** 2025-09-01

**Authors:** Dipta Sumeru Rinandio, Iin Pertiwi A. Husaini, Mahat Magandhi, Irfan Martiansyah, Tri Yuni Indah Wulansari, Julisasi Tri Hadiah, Deden Girmansyah, Nanda Utami, Rugayah, Arifin Surya Dwipa Irsyam, Rina Ratnasih Irwanto, Agus Suhatman, Muhammad Rifqi Hariri

**Affiliations:** ^1^ Research Center for Ecology and Ethnobiology, National Research and Innovation Agency, Cibinong, Bogor, Indonesia; ^2^ Research Center for Applied Botany, National Research and Innovation Agency, Cibinong, Bogor, Indonesia; ^3^ Department of Biology, Faculty of Mathematic and Natural Sciences, Universitas Indonesia, Depok, Indonesia; ^4^ Research Center for Biosystematics and Evolution, National Research and Innovation Agency, Cibinong, Bogor, Indonesia; ^5^ Herbarium Bandungense, School of Life Sciences and Technology, Institut Teknologi Bandung, Bandung, Indonesia; ^6^ Directorate for Scientific Management Collection, National Research and Innovation Agency, Cibinong, Bogor, Indonesia

**Keywords:** Celastrales, gene annotation, genome structure, illumina, plastome assembly

## 1 Introduction

*Elaeodendron glaucum* (Rottb.) Pers., a woody species in the family Celastraceae, is widely distributed across Sri Lanka to South East Asia Hou, 1960; (POWO, 2025). Despite its broad geographic range and presumed ecological adaptability, genomic data for this species remain scarce (Simmons et al., 2012). In recent years, chloroplast genome characterization has emerged as a powerful tool for resolving phylogenetic relationships and elucidating evolutionary patterns in angiosperms (Zhang and Ma, 2024). For example, a recent comparative analysis of complete chloroplast genomes from 13 species of the genus *Celastrus* successfully reconstructed a well-supported phylogenetic tree, clarified relationships within the genus, and confirmed that *Celastrus* forms a monophyletic group with *Tripterygium* as its closest sister lineage. The comparative genomic analysis pinpointed distinct variable regions suitable as molecular markers for species delimitation and demonstrated that *C. tonkinensis* Pit. and *C. hindsii* Benth. are conspecific (Liu et al., 2024).

Chloroplast genomes are particularly valuable due to their highly conserved structure, maternal inheritance, and moderate mutation rates, which make them effective molecular markers for species identification, comparative genomics, and systematic classification (Daniell et al., 2016; Nadeem et al., 2018). In this context, the complete sequencing and structural annotation of the *E. glaucum* chloroplast genome addresses a notable gap in genomic resources and offers a critical foundation for improving taxonomic resolution within the Celastraceae (Coughenour et al., 2010). This research is especially relevant given the persistent taxonomic ambiguities within the genus *Elaeodendron* Jacq., which have been confounded by morphological convergence and a lack of comprehensive molecular data (Simmons et al., 2012). By generating and analyzing the complete chloroplast genome of *E. glaucum*, the present study provides essential data to clarify phylogenetic relationships both within the genus and among closely related taxa. Comparative analyses with other chloroplast genomes may further reveal species-specific structural variations, genomic signatures, and adaptive traits, thereby contributing to a more refined and reliable classification framework for the family.

The chloroplast genome in most land plants is characterized by a conserved quadripartite circular structure comprising a large single-copy (LSC) region, a small single-copy (SSC) region, and two inverted repeat (IR) regions (Dobrogojski et al., 2020). However, structural exceptions such as the loss of IRs or SSCs have been reported (Wang et al., 2024). The overall size of chloroplast genomes typically ranges from approximately 19 to 217 kilobases, with IR regions spanning 20 to 26 kilobases (NCBI Organelle Genome Resources). The chloroplast proteome includes roughly 3,000 proteins involved in crucial metabolic pathways, including photosynthesis, as well as the biosynthesis of fatty acids, amino acids, nucleotides, vitamins, hormones, and secondary metabolites (Dobrogojski et al., 2020). Most of these proteins are encoded by nuclear genes, synthesized in the cytosol, and subsequently imported into the chloroplast, while a smaller fraction is encoded by the chloroplast genome itself (Fu et al., 2022).

Recent advancements in chloroplast genome engineering have facilitated detailed investigations into gene function, regulatory mechanisms, and targeted genome modification (An et al., 2022). These technologies are increasingly being applied to enhance photosynthetic performance, develop nutritionally improved crops, and produce high-value bioproducts (Daniell et al., 2021; Singhal et al., 2023). This study reports the *de novo* sequencing, annotation, and structural characterization of the complete chloroplast genome of *E. glaucum*, presenting a valuable genomic reference for future research in taxonomy, evolutionary biology, and biotechnological innovation.

## 2 Method

### 2.1 Plant material

Fresh leaf material of *E. glaucum* was collected from a cultivated individual maintained at the Kebun Raya Bogor (Bogor Botanic Gardens), West Java, Indonesia, under accession number III.G.189. The specimen’s original provenance is traced to Puger, East Java.

### 2.2 DNA extraction

Genomic DNA was extracted from young leaf tissue using a modified cetyltrimethylammonium bromide (CTAB) protocol following Doyle and Doyle (1987), with optimizations implemented to enhance yield and purity. DNA quality and quantity were initially assessed using a NanoDrop 2000 spectrophotometer (Thermo Scientific), and integrity was evaluated via 1% TBE agarose gel electrophoresis. For more accurate quantification, the Qubit dsDNA High Sensitivity Assay Kit (Thermo Scientific) was used. Fragment size distribution and integrity were further validated using the Agilent 4150 TapeStation system.

### 2.3 Whole genome sequencing

High-quality genomic DNA was subsequently subjected to library preparation. The DNA was enzymatically fragmented to produce insert-sized fragments appropriate for high-throughput sequencing. Following fragmentation, sequencing libraries were constructed and sequenced on the Illumina NextSeq 500 platform (Genetika Science Lab, Tangerang, Indonesia), generating paired-end reads of 150 base pairs. The sequencing run targeted a total yield of 10 gigabases, providing sufficient depth for comprehensive chloroplast genome assembly and downstream analyses.

### 2.3 Chloroplast genome assembly and annotation

Quality assessment of the raw sequencing reads was conducted using FastQC version 0.11.8 (Andrews, 2010), which provided diagnostic metrics including per-base quality scores, GC content, sequence length distribution, and indicators of potential contamination. To ensure high-fidelity reads, adapter sequences, low-quality bases (Phred score <30), and nucleotide biases at the 5′ and 3′ ends were removed using Trimmomatic version 0.39 (Bolger et al., 2014). The following trimming parameters were applied: ILLUMINACLIP: TruSeq3-PE.fa:2:30:10, SLIDINGWINDOW:4:28, LEADING:28, TRAILING:28, and MINLEN:20. These processes were executed through the Galaxy web platform (https://usegalaxy.org, The Galaxy Community, 2024).

High-quality trimmed reads were assembled *de novo* into a complete chloroplast genome using GetOrganelle version 1.7.7.1 (Jin et al., 2020), an organelle-specific assembler employing a k-mer-based graph approach optimized for high-coverage plastid genomes. Genome annotation was performed using CPGAVAS2 (Shi et al., 2019) through its online platform (http://47.96.249.172:16019/analyzer/annotate), with the chloroplast genome of *Euonymus kiautschovicus* Loes. (syn. *Euonymus fortunei* var. *fortunei*, GenBank accession: PQ397793) serving as the reference to guide gene prediction and structural feature identification.

To ensure annotation precision, subsequent manual curation and validation of coding sequences, intron-exon boundaries, and RNA genes were carried out using Unipro UGENE version 45.1 (Okonechnikov et al., 2012) and NCBI Genome Workbench version 3.8.2 (Kuznetsov and Bollin, 2021). Finally, the complete circular chloroplast genome map was visualized using OrganellarGenomeDRAW (OGDRAW) through the MPI-MP Chlorobox web server (Greiner et al., 2019), enabling clear graphical representation of gene content, orientation, and overall genome architecture.

## 3 Data

### 3.1 Characterization of *Elaeodendron glaucum* chloroplast genome

**Figure 1:**
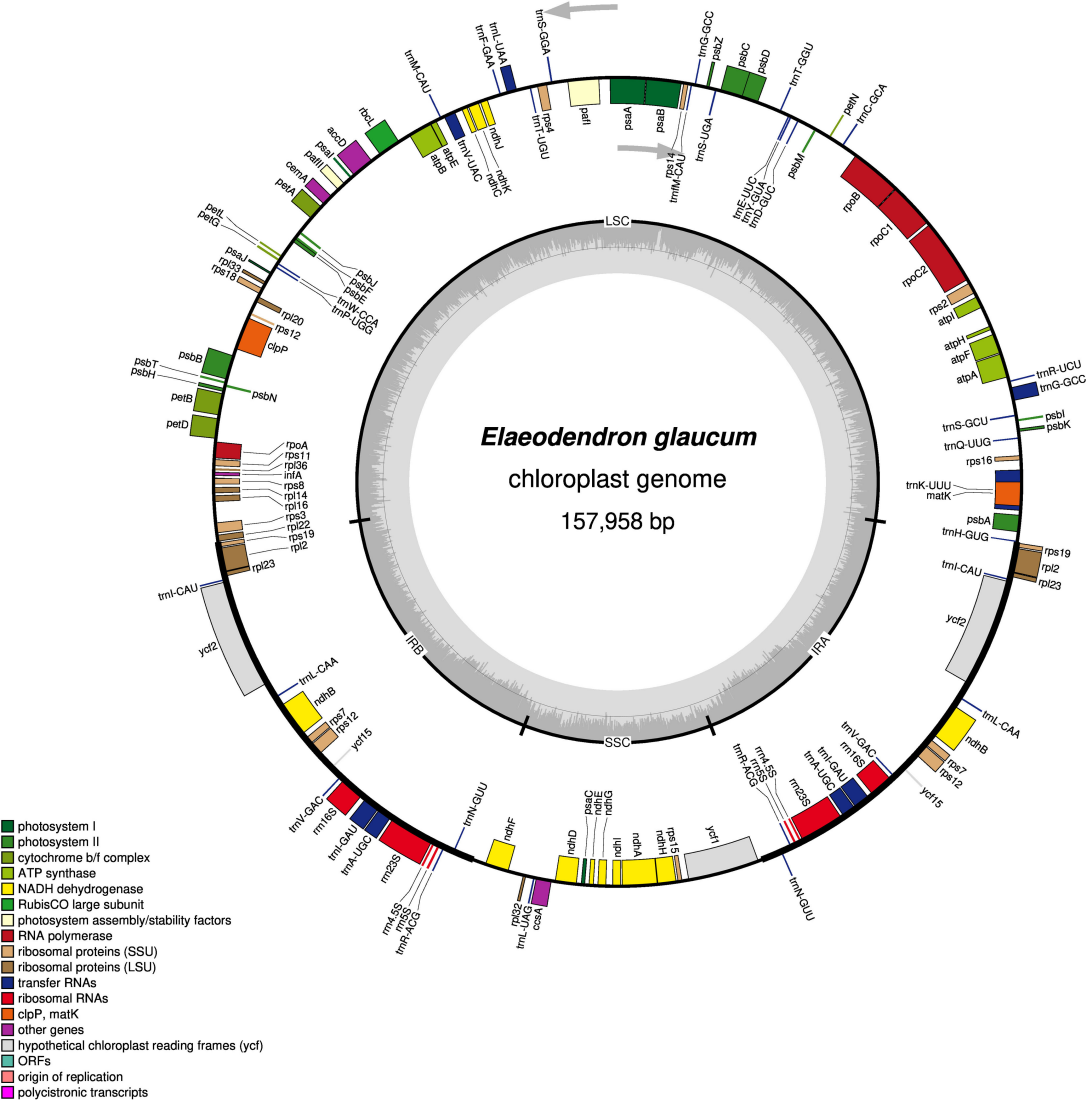
Circular gene map of the *Elaeodendron glaucum* chloroplast genome. Genes positioned on the inner track of the circular map are transcribed in the counterclockwise direction, while those on the outer track are transcribed clockwise. Distinct colors are used to indicate different functional categories of genes. The innermost circle illustrates the GC content in grey, with the lighter grey areas representing AT content.

The complete chloroplast genome of *E. glaucum* was assembled as a circular molecule of 157,958 base pairs (bp) with an overall GC content of 37%. Its organization follows the typical quadripartite structure of angiosperm plastomes, consisting of a large single-copy (LSC) region of 86,485 bp with 35.21% GC content, a small single-copy (SSC) region of 18,363 bp with 31.80% GC content, and two inverted repeat (IR) regions of 26,555 bp each with 42.79% GC content (**Figure 1**). The total genome size of *E. glaucum* is slightly larger than those reported for closely related genera within the Celastraceae family, including *Celastrus vaniotii* (H.Lév.) Rehder (157,194 bp; GenBank accession: OR726632), *E. kiautschovicus* (157,611 bp; GenBank accession: PQ397793), *Microtropis osmanthoides* (Hand.-Mazz.) Hand.-Mazz. (156,659 bp; GenBank accession: NC 065714), and *Parnassia faberi* Oliv. (153,846 bp; GenBank accession: NC 061028), but shorter than *Salacia menglaensis* J.Y.Shen, L.C.Yan & Landrein (163,255 bp; GenBank accession: NC 047214).

### 3.2 Gene annotation of *Elaeodendron glaucum* chloroplast genome

A total of 133 genes were annotated within the *E. glaucum* chloroplast genome, encompassing 112 unique genes. These include 88 protein-coding genes (79 unique), 37 transfer RNA (*t*RNA) genes (29 unique), and 8 ribosomal RNA (*r*RNA) genes (4 unique). Among these, 17 genes contain a single intron, while three genes—*rps12*, *pafI*, and *clpP*—each possess two introns (**Table 1**).

**Table 1:** List of genes in the *Elaeodendron glaucum* chloroplast genome.

Functional category	Group of Gene	Name of Gene
Self-replication	rRNA	*rrn16^d^, rrn23^d^, rrn4.5^d^, rrn5^d^*
	tRNA	trnH-GUG, trnK-UUU^*^, trnQ-UUG, trnS-GCU, trnG-GCC^d*^, trnR-UCU, trnC-GCA, trnD-GUC, trnY-GUA, trnE-UUC, trnT-GGU, trnS-UGA, trnfM-CAU^d^, trnS-GGA, trnT-UGU, trnL-UAA^*^, trnF-GAA, trnV-UAC^*^, trnW-CCA, trnP-UGG, trnI-CAU^d^, trnL-CAA^d^, trnV-GAC^d^, trnI-GAU^d*^, trnA-UGC^d*^, trnR-ACG^d^, trnN-GUU^d^, trnL-UAG
	Large subunit ribosomal proteins (LSU)	rpl14, rpl16, rpl2^d*^, rpl20, rpl22, rpl23^d^, rpl32, rpl33, rpl36
	Small subunit ribosomal proteins (SSU)	rps11, rps12^d**^, rps14, rps15, rps16, rps18, rps19 ^d^, rps2, rps3, rps4, rps7 ^d^, rps8
	DNA dependent RNA polymerase	rpoA, rpoB, rpoC1^*^, rpoC2
	Subunits of ATP synthase	atpA, atpB, atpE, atpF^*^, atpH, atpI
	Subunits of NADH-dehydrogenase	ndhA^*^, ndhB^d^*, ndhC, ndhD, ndhE, ndhF, ndhG, ndhH, ndhI, ndhJ, ndhK
Photosynthesis	Subunits of photosystem I	*psaA, psaB, psaC, psaI, psaJ*
	Subunits of photosystem II	psbA, psbB, psbC, psbD, psbE, psbF, pdbH, psbI, psbJ, psbK, psbM, psbN, psbT, psbZ, pafI^**^
	Subunits of cytochrome b/f complex	petA, petB*, petD*, petG, petL, petN
	Subunit rubisco	*rbcL*
	Subunit of acetyl-CoA-carboxylase	*accD*
	C-type cytochrome synthesis gene	*ccsA*
Other function	Protease	clpP^**^
	Maturase	*matK*
	Envelope membrane protein Translational initiation factor	*cemA* *infA*
Unknown function	Conserved open reading frames	ycf1^*^, ycf15^d^, ycf2^d^, pafII

d, gene duplication; *, single intron; **, double intron.

## Data Availability

The datasets presented in this study can be found in online repositories. The names of the repository/repositories and accession number(s) can be found below: NCBI GenBank, accession PV153503.
